# Epitranscriptomics: A New Layer of microRNA Regulation in Cancer

**DOI:** 10.3390/cancers13133372

**Published:** 2021-07-05

**Authors:** Veronica De Paolis, Elisa Lorefice, Elisa Orecchini, Claudia Carissimi, Ilaria Laudadio, Valerio Fulci

**Affiliations:** Dipartimento di Medicina Molecolare, Sapienza Università di Roma, 00161 Rome, Italy; veronica.depaolis@uniroma1.it (V.D.P.); elisa.lorefice@uniroma1.it (E.L.); elisa.orecchini@uniroma1.it (E.O.); valerio.fulci@uniroma1.it (V.F.)

**Keywords:** microRNA, cancer, epitranscriptomics, m6A, m5C, A-to-I editing, m7G

## Abstract

**Simple Summary:**

MicroRNAs are small non-coding RNAs, acting as post-transcriptional regulators of gene expression. In the last two decades, their role in cancer as oncogenes (oncomir), as well as tumor suppressors, has been extensively demonstrated. Recently, epitranscriptomics, namely the study of RNA modifications, has emerged as a new field of great interest, being an additional layer in the regulation of gene expression. Almost all classes of eukaryotic RNAs, including miRNAs, undergo epitranscriptomic modifications. Alterations of RNA modification pathways have been described for many diseases—in particular, in the context of malignancies. Here, we reviewed the current knowledge on the potential link between epitranscriptomic modifications of miRNAs and cancer.

**Abstract:**

MicroRNAs are pervasive regulators of gene expression at the post-transcriptional level in metazoan, playing key roles in several physiological and pathological processes. Accordingly, these small non-coding RNAs are also involved in cancer development and progression. Furthermore, miRNAs represent valuable diagnostic and prognostic biomarkers in malignancies. In the last twenty years, the role of RNA modifications in fine-tuning gene expressions at several levels has been unraveled. All RNA species may undergo post-transcriptional modifications, collectively referred to as epitranscriptomic modifications, which, in many instances, affect RNA molecule properties. miRNAs are not an exception, in this respect, and they have been shown to undergo several post-transcriptional modifications. In this review, we will summarize the recent findings concerning miRNA epitranscriptomic modifications, focusing on their potential role in cancer development and progression.

## 1. Introduction

MicroRNAs (miRNAs) are a class of short, non-coding RNAs that control gene expression at the post-transcriptional level via either translational repression or mRNA degradation.

Since miRNAs act as pervasive regulators of gene expression, it is not surprising that they were involved in normal animal development and in a variety of biological processes [1,2]. The aberrant expression of miRNAs is also associated with many human diseases [3,4].

A large amount of literature documents the wide involvement of miRNAs in cancer as key players in the development and progression of different malignancies (reviewed in reference [5]), as diagnostic/prognostic biomarkers (reviewed in reference [6]) and as potential therapeutic targets [7].

One hundred and seventy-two post-transcriptional modifications of RNAs have been reported thus far [8], collectively known as the “epitranscriptome” [9]. Some of these epitranscriptomic modifications have been thoroughly investigated, unraveling their contribution to RNA stability and/or activity [10,11,12]. The most common and best-characterized epitranscriptomic modifications include N6-methyl-Adenosine (m6A) [13], pseudoUridine (Ψ) [14], Adenosine-to-Inosine (A-to-I) editing [15] and 5-methyl-Cytidine (m5C) [16].

In epigenetics, a widely exploited paradigm postulates that DNA methylation and histone modifications are installed by “writer” enzymes, recruit “reader” proteins and are removed by “eraser” enzymes [17]. Although it has been proposed that the same general view may hold true for epitranscriptomic modifications, the intrinsic features of RNA imply that “readers” and “erasers” may be dispensable for some modifications [18]. “Writer” enzymes have been identified for all major RNA modifications [19,20,21,22,23,24]. Otherwise, “reader” proteins have been described only for m6A [25] and m5C [26]. Several RNA modifications directly affect the RNA structure and/or base pairing, thus requiring no “reader” proteins to exert their functions. This is obvious for A-to-I editing, which changes the identity of a base, and it has also been demonstrated for Ψ [27,28]. Furthermore, while it has been suggested that m6A can be removed from modified RNA molecules [29,30], most epitranscriptomic modifications are apparently not dynamic. On the one hand, because of the very short half-life of most eukaryotic RNAs, specific “eraser” enzymes might be dispensable at least for some epitranscriptomic modifications that may actually be removed through the rapid turnover of modified RNA molecules. On the other hand, epitranscriptomic modifications on more stable RNA molecules (e.g., rRNAs) may lack any “eraser” enzymes simply because reverting such modifications is not beneficial to the cell. Accordingly, no “eraser” enzyme has been identified yet for m5C, Ψ, A-to-I editing and many other epitranscriptomic modifications [31,32].

The first evidence of an epitranscriptomic modification in miRNAs was reported in 2004 [33]. From that moment on, the role of the epitranscriptomics of miRNAs in cancer promotion and progression started to be elucidated. Notably, epitranscriptomic modifications have also been described in RNAs targeted by miRNAs, positively or negatively affecting miRNA:target interactions.

In this review, we will focus on the current evidence supporting the role in cancer of epitranscriptomic modifications of miRNAs and of miRNA-targeted RNAs.

## 2. miRNAs: Biogenesis and Functions

miRNAs are a class of small (18–24 nt) non-coding RNAs that are processed from long primary miRNAs (pri-miRNAs) generally transcribed by RNA Polymerase II [34,35,36] and harbor one or more hairpin structure [37]. Pri-miRNA processing starts in the nucleus, where the Microprocessor complex, formed by the RNase III enzyme DROSHA, the RNA-binding protein Di George Syndrome Critical Region Gene 8 (DGCR8) and other proteins [38,39] catalyzes the endonucleolytic cleavage of the pri-miRNA to yield a ~70-nt-long hairpin pre-miRNA [40].

Pre-miRNAs are then exported to the cytoplasm by Exportin-5 [41,42,43]. In the cytoplasm, pre-miRNAs undergo further cleavage by DICER, which removes the terminal loop of the hairpin to yield a duplex consisting of the mature miRNA (guide strand) base-paired to the passenger strand [44,45].

Mature miRNAs interact with the RNA-binding proteins belonging to the Argonaute family (AGO), thus becoming integral components of the RNA-Induced Silencing Complex (RISC) (reviewed in references [46,47]). The mature miRNA within RISC recruits the complex onto target RNA molecules by base-pairing between a “seed” region (nt 2–7) at the 5′ end of the miRNA and the 3′ UTR of the target RNA [48,49,50], leading to gene silencing through translation repression and mRNA decay.

MiRNA expression is tightly controlled in cells by mechanisms acting at both the transcriptional and post-transcriptional levels (reviewed in references [51,52,53]). The titration of miRNAs by competing endogenous RNAs (ceRNAs) adds a further layer of regulation of miRNA activity [54,55,56,57]. These long RNA molecules (which can be mRNAs, lncRNAs, pseudogene-encoded RNAs or circRNAs) sequester miRNAs, thereby preventing their interaction with other targets, whose repression is therefore relieved.

MiRNAs participate in gene regulatory networks that control diverse biological processes in multicellular organisms, such as animal development (reviewed in reference [1]), cell fate specification and differentiation [58], the immune response [59] and inflammation [60]. Changes in the miRNA expression levels have been associated with a wide range of human diseases, including diabetes, cardiovascular and kidney disease and cancer [3,4]. A huge number of miRNAs are downregulated or upregulated in human cancers, where they exert oncogenic or tumor suppressor functions, depending on the cellular context. Alterations of miRNAs in different malignancies have been linked to genetic deletion or amplification, as well as to DNA methylation of the miRNA genomic loci, to the modulation of the pri-mRNA transcription level by transcription factors or to the dysregulation of one or more steps in miRNA biogenesis (reviewed in reference [5]). Recently, epitranscriptomics is emerging as an additional layer of the regulation of the miRNA function in cancer.

## 3. Epitranscriptomic Modifications of miRNA in Cancer

### 3.1. N6-Methyl-Adenosine (m6A)

m6A was first reported in the 1970s in mammalian RNAs [61,62,63]. A full comprehension of the role of this modification took several decades. In 1997, the protein Methyltransferase-like (METTL) 3 was identified as the first “writer” of m6A in mammalian cells [23]. Further investigations have shown that m6A is installed by a nuclear complex comprised of METTL3, METTL14 and WT1-Associated Protein (WTAP) [22]. Further components of this complex include KIAA1429, RNA Binding Motif Protein 15 (RBM15) and Zinc Finger CCCH-Type Containing 13 (ZC3H13) [64,65,66].

Several members belonging to the YTH (YT521-B homology) family, such as human YT521-B (also known as YTHDC1), YTHDC2, YTHDF1, YTHDF2 and YTHDF3, have been identified as m6A-binding or “reader” proteins [18,25]. The members belonging to the DF family likely confine m6A-modified RNAs in specific cytoplasmic liquid–liquid phase separation compartments [67].

Several other “reader” proteins have been shown to bind m6A-modified RNAs thanks to a so-called “m6A switch” [68]. Indeed, m6A installation may trigger a conformational switch that allows the binding of these “reader” proteins, which, in fact, do not directly bind to the m6A residue itself [13]. This mechanism is exploited by several members of the hnRNP (heterogeneous nuclear ribonucleoprotein) family. Finally, insulin-like growth factor 2 mRNA-binding proteins (IGF2BP) were also reported to bind m6A-modified RNAs, promoting their stability [69].

Although two enzymes able to “erase” m6A from mammalian RNAs have been reported, i.e., FTO Alpha-Ketoglutarate-Dependent Dioxygenase (FTO) and AlkB Homolog 5, RNA Demethylase (ALKBH5) [29,30], the specificity and the relevance of these enzymes in physiological conditions are still a matter of debate [70].

About 0.1–0.4% of all adenosines in global cellular RNAs are modified as m6A, and this modification accounts for ~50% of all methylated ribonucleotides [61]. m6A was found in all classes of cellular RNAs: mRNAs (in particular, in long internal exons, locations upstream of stop codons and the 3′-UTR regions) [25,71,72]; ribosomal RNAs; transfer RNAs and various non-coding RNAs [73,74,75].

The first report of m6A modification in miRNAs dates back to 2014, when Yuan and colleagues [76] reported this epitranscriptomic modification in miR-125b. Surprisingly, the authors identified NOP2/Sun RNA Methyltransferase 2 (Nsun2), a well-characterized m5C “writer” [21,77], as the “writer” enzyme of this modification. Furthermore, their data suggested that a m6A modification may prevent pri-miR-125b-2 processing into mature miR-125b [76].

In 2015, two pivotal contributions by Alarcón and colleagues [78,79] provided evidence that a m6A modification by METTL3 globally enhances miRNA processing in mammalian cells. Mechanistically, a novel nuclear m6A “reader”, namely hnRNPA2B1, binds to m6A-modified pri-miRNAs and interacts with the Microprocessor complex, promoting miRNA processing in a METTL3-dependent manner. Interestingly in *Arabidopsis thaliana*, an ortholog of METTL3 has been recently shown to mediate m6A installation on pri-miRNAs, thus promoting an interaction with the Microprocessor complex, suggesting that the regulation of miRNA processing by m6A is a widely conserved mechanism [80].

Neither FTO nor ALKBH5 were proven yet to be able to catalyze the demethylation of miRNAs. However, it is worth mentioning that DEAD-box RNA helicase 3 (DDX3) controls the methylation status of microRNAs and interacts with both the AGO2 and the ALKBH5 enzymes [81], suggesting a possible role of ALKBH5 in miRNA demethylation.

In cancer, the relevancy of m6A in miRNA maturation was first unveiled for miR-126 in hepatocellular carcinoma (HCC) [82]. The authors showed that a METTL14-dependent m6A modification on pri-miR-126 is required for its processing by the Microprocessor complex. Indeed, in hepatocellular carcinoma, METTL14 is downregulated, thus decreasing m6A on pri-miR-126 and reducing the miR-126 abundance. Furthermore, their findings support the hypothesis that the miR-126 modification by METTL-14 is instrumental to prevent cell invasion, as assessed by in vitro assays.

**Figure 1:**
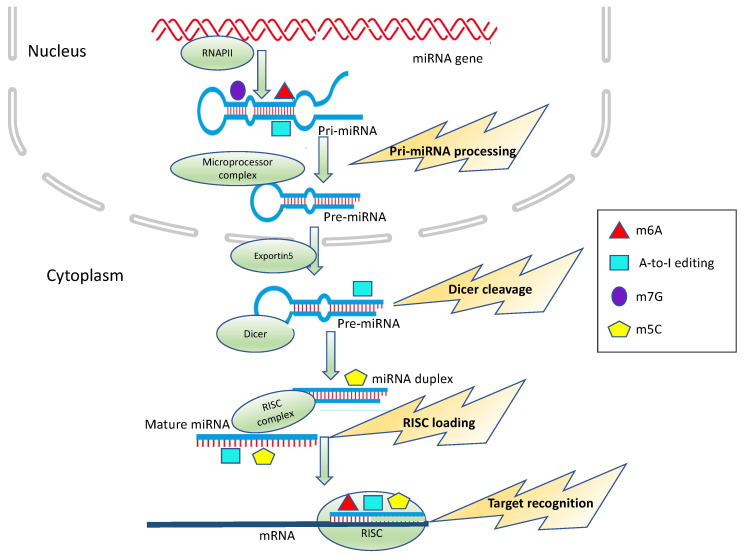
Epitranscriptomic modification impacts on miRNA processing and activity. m6A, A-to-I editing, m5C and m7G can affect different steps of miRNA biogenesis, including Microprocessor cleavage, Dicer cleavage and RISC loading or alter target recognition and binding.

From that moment on, increasing evidence has disclosed the relevance of the m6A modification of miRNA in cancer progression. Most of the literature confirms that m6A mainly promotes pri-miRNA processing and that the deregulation of the enzymes involved in writing or reading m6A is correlated with tumor onset. Notably, alteration of the m6A deposition on miRNAs is not only a common feature of different tumors but also participates in tumorigenesis processes (Figure 1 and Table 1).

Colorectal cancer (CRC) displays a low expression of METTL14, and this is associated with an impaired m6A modification and processing of pri-miR-375. Low levels of miR-375 result in the overexpression of its targets YAP1 (Yes-associated protein 1) and SP1, thus increasing cell proliferation, migration and invasion [83].

The overexpression of METTL3 in bladder, colorectal, lung, ovarian and gallbladder cancers promotes the processing of several oncomirs, such as miR-221/222 [84], miR-1246 [85], miR-143 [86], miR-126 [87] and miRNA-92 [88]. These miRNAs have tumor suppressors as targets; therefore, the m6A-dependent accumulation of miRNAs results in promoting tumor progression. The same mechanism was described by Zhang and colleagues [89], who recently showed that, in response to cigarette smoke condensate in pancreatic ductal adenocarcinoma, there was an accumulation of mature miR-25-3p, caused by the overexpression of METTL3, which enhanced the m6A modification of pri-miR-25-3p. Increased miR-25-3p resulted in the repression of its target PH Domain and Leucine-Rich Repeat Protein Phosphatase 2 (PHLPP2), thus triggering AKT activation. Interestingly, a novel m6A reader, namely NF-κB-Associated Protein (NKAP), facilitated the interaction of pri-miR-25 with DGCR8, thus promoting the maturation of miR-25-3p [89].

A recent report identified a further putative m6A “reader” protein, RALY (also known as hnRNPCL2), which interacts with miR-483, miR-676 and miR-877 in CRC and is required for their m6A-dependent processing. Mechanistically, RALY interacts with DROSHA and DGCR8 to enhance pri-miRNA processing [90]. 

Recently, it has been proposed that a m6A modification reduces the ability of miRNAs to suppress target mRNA translation. Indeed, Konno and colleagues [91] showed that the m6A modification of let-7a-5p and miR-17-5p caused a large structural change in the RISC complex, which affected the target RNA recognition. In pancreatic and CRC tissues, the m6A levels on let-7a-5p and miR-17-5p increased without affecting the miRNA expression level.

**Table 1:** Effects of m6A modification of miRNAs in cancer.

Cancer Type	m6A-Modified miRNA(s)	Increase/ Decrease ^1^	Effects on miRNA Processing/Function	Effects on Tumor Progression	Reference
Colorectal Cancer	miR-1246	↑	processing	Up-regulation of mature of miR-1246 results in the reduction of SPRED2, thus activating the RAF/MEK/ERK pathway	[85]
	miR-375	↓	processing	Down-regulation of mature miR-375 increases the expression of its targets YAP1 and SP1 thus increasing proliferation, and migration and invasion	[83]
	miR-483, miR-676 miR-877	n.d.	processing	miR-483, miR-676 and miR-877 modulate mitochondrial metabolism by targeting electron transport chain genes	[90]
	miR-17-5p let-7a-5p	↑	Binding to targets	n.d.	[91]
Pancreatic cancer	miR-25-3p	↑	processing	Up-regulation of mature miR-25-3p results in the reduction of PHLPP2, leading to AKT activation.	[89]
	miR-17-5p let-7a-5p	↑	Binding to targets	n.d.	[91]
Hepatocellular Carcinoma	miR-126	↓	processing	Down-regulation of mature miR-126 which acts as a tumor suppressor	[82]
Bladder cancer	miR-221/222	↑	processing	Up-regulation of mature miR-221/222 results in the reduction of PTEN, leading to proliferation	[84]
Gallbladder cancer	miRNA-92	↑	processing	Up-regulation of mature miRNA-92 results in the reduction of PTEN, thus activating PI3K/AKT signaling	[88]
Ovarian cancer	miR-126	↑	processing	Up-regulation of mature miR-126-5p results in the reduction of PTEN, thus activating the PI3K/Akt/mTOR pathway	[87]
Gastric cancer	miR-17-5p let-7a-5p	↑	Binding to targets	n.d.	[91]
Lung cancer (brain metastasis)	miR-143-3p	↑	processing	Up-regulation of mature miR-143-3p promotes the metastatic potential of lung cancer via regulation of angiogenesis and microtubules through VASH1	[86]

^1^ increase (↑) or decrease (↓) of the epitranscriptomic modification (n.d., not detected; *SPRED2*, Sprouty Related EVH1 Domain Containing 2; *YAP1*, yes-associated protein 1; *SP1*, Sp1 Transcription Factor; *PHLPP2*, PH Domain And Leucine Rich Repeat Protein; *PTEN*, Phosphatase 2 Phosphatase And Tensin Homolog; *VASH1*, Vasohibin 1.

### 3.2. A-to-I Editing

A-to-I editing is catalyzed by enzymes highly conserved in vertebrates, called Adenosine Deaminases Acting on RNA (ADAR) [92]. Mammalian genomes encode for three members of the ADAR family: ADAR1, ADAR2 and ADAR3 [93].

ADAR enzymes bind double-stranded (ds) regions of coding and noncoding RNAs [94]; in RNAs forming imperfect dsRNA structures, A-to-I editing involves only one or two adenosines (site selective editing), while, in the case of long perfect dsRNA regions, the random modification of several A residues is observed (hyper-editing) [95,96,97].

Inosine is recognized by the cellular machinery as guanosine, causing a change in the RNA sequence. As a consequence, depending on the modification site, this type of RNA editing can influence the RNA stability [98,99,100], splicing [101,102,103], localization and translation, as well as redefine its interactions with specific factors [104,105]. In mRNAs, the modification of A-to-I can lead to a codon change, thus affecting the primary structure of the encoded protein [106,107].

A-to-I editing mainly targets noncoding regions of RNA, such as introns and UTRs, containing repetitive Alu elements and Long Interspersed Elements (LINEs) that fold into dsRNA structures recognized by ADARs [108].

In most types of cancer, the activity of ADAR enzymes is significantly decreased, as witnessed by the extensive hypoediting of Alu RNAs, as well as by the reduced expression of ADAR enzymes [109].

The first evidence of the editing of a miRNA was shown in 2004 by Luciano and colleagues [33], who reported A-to-I conversion within the miR-22 precursor in *Homo sapiens* and *Mus musculus*. Soon after, it was shown that the A-to-I editing of pri-miR-142 prevents processing by DROSHA [110]. ADAR enzymes have a degree of specificity for different miRNA precursors, depending on their secondary structure [111]. The ADAR1 interaction with DICER was associated with enhanced miRNA processing in oral squamous cells carcinoma [112] and in melanoma [113], although, in both cases, the authors did not assess the editing of the miRNA precursors. Furthermore, ADAR editing has been shown to affect the DICER-dependent processing of viral miRNAs [114]. Of note, ADARs can also alter miRNA metabolism independently from their editing activity [115,116,117].

Several examples showed that the A-to-I editing of miRNA precursors inhibits the biogenesis of mature miRNAs (Figure 1 and Table 2). The deregulation of ADAR1 and/or ADAR2 in glioblastoma and in chordoma affects the expression levels of miR-21, miR-221 and miR-222 [118] and of miR-10a and miR-125a [119], respectively. Furthermore, the impairment of let-7 biogenesis by means of ADAR1-mediated A-to-I editing drives leukemia stem cells renewal [120].

A further mechanism by which A-to-I editing alters miRNA functions is the remodulation of potential targets of mature miRNAs [121]. Indeed, A-to-I editing in the “seed” sequence causes a loss-of-function when no more targets are recognized [122] or a gain-of-function when a new target is recognized by the edited miRNA [123,124]. Therefore, changes in the relative abundance of the edited and unedited forms of the miRNA lead, in turn, to altered gene expression profiles.

In this context, in 2012, it has been shown that the loss of mir-376a-5p editing results in the increased invasiveness of glioblastoma multiforme (GBM). Mechanistically, unedited miR-376a-5p promotes aggressive glioma growth by its ability to target Ras-Related Protein Rap-2a (RAP2A), a member of the RAS oncogene family, and the concomitant inability to target Autocrine Motility Factor Receptor (AMFR) [125]. These findings were further corroborated by the discovery that miRNA hypoediting is widespread in GBM [126].

Similarly, the impairment of ADAR-mediated editing of miR-455-5p enhances the progression in melanoma. Indeed, unedited miR-455-5p targets the tumor suppressor Cytoplasmic Polyadenylation Element-Binding Protein 1 (CPEB1), thus promoting metastasis, while edited miR-455-5p exerts the opposite effect [127]. In a follow-up of their work, the authors showed that, in melanoma, the editing of miR-378a-3p allows the targeting of the oncogene Parvin Alpha (PARVA). Hence, the loss of miR-378a-3p editing promotes melanoma progression [128].

In the brain, ADAR2 edits the seed sequence of miR-589-3p. In glioblastoma, the editing of miR-589-3p decreases. Higher levels of the unedited version of miR-589-3p promote proliferation and invasion by targeting the tumor suppressor Protocadherin 9 (PCDH9). On the contrary, editing within miR-589-3p retargets the miRNA to the ADAM Metallopeptidase Domain 12 (ADAM12) to contrast the progression of the tumor [129].

In different contexts, miRNA A-to-I editing stimulates the progression of the tumor by altering the selection of miRNA targets. In thyroid cancer, the slight overexpression of ADAR1 corresponds to a higher expression of ZEB1, a master regulator of Epithelial–Mesenchymal Transition (EMT). It has been demonstrated that editing of the seed sequence of miR-200b by ADAR1 impairs its ability to inhibit ZEB1 expression, favoring the progression of the cancer [130,131].

Another target of A-to-I editing is miR-381, a microRNA involved in stemness and chemoresistance [132,133] that is overedited in non-small cell lung carcinoma (NSCLC) cell lines harboring the genomic amplification of ADAR1. Edited miR-381 promotes cell viability [134].

Besides these examples on specific miRNAs, a global analysis of miRNA sequencing data from healthy and cancerous tissues unveiled that miRNA editing is frequently dysregulated in cancer [130,135,136,137,138,139].

**Table 2:** Effects of A-to-I editing of miRNAs in cancer.

Cancer	A-to-I-Modified miRNA(s)	Increase/ Decrease ^1^	Effects on miRNA Processing/Function	Effects on Tumor Progression	Reference
Glioma	mir-376a-5p	↓	Binding to targets	Unedited miR-376a-5p promotes aggressive glioma growth, by its ability to target RAP2A and concomitant inability to target AMFR	[125]
	miR-221/222 miR-21	↓	processing	Up-regulation of mature miR-221/222 and miR-21 results in the repression of its targets p27Kip1 and PDCD4, thus increasing proliferation and migration of glioblastoma	[118]
	miR-589-3p	↓	Binding to targets	Editing within miR-589–3p retargets the miRNA from the protocadherin PCDH9 to the metalloprotease ADAM12, which is involved in glioblastoma cell invasion.	[129]
Melanoma	miR-455-5p	↓	Binding to targets	Unedited miR-455-5p but not the edited form targets the tumor suppressor gene CPEB1, thus promoting tumor growth and metastasis	[127]
	miR-378a-3p	↓	Binding to targets	Edited miR-378a-3p but not the unedited form specifically targets the PARVA oncogene, thus preventing the progression of melanoma towards the malignant phenotype	[128]
Chordoma	miR-10a miR-125a	↑	processing	Down-regulation of miR-10a and miR-125a expression and upregulates expression of their target genes	[119]
Chronic myeloid leukemia	let-7	↑	processing	Down-regulation of mature let-7 results in increased LIN28B expression and enhanced self-renewal	[120]
Thyroid cancer	miR-200b	↑	Binding to targets	Edited miR-200b has weakened activity against its target gene ZEB1, an epithelial–mesenchymal transition (EMT) marker	[131]
Lung cancer	miR-381	↑	n.d.	Edited miR-381 enhances the growth of non-small-cell lung cancer cells as compared to the unedited form	[134]

^1^ increase (↑) or decrease (↓) of the epitranscriptomic modification (n.d., not detected; *RAP2A*, Ras-Related Protein Rap-2a; *AMFR*, Autocrine Motility Factor Receptor; *PDCD4*, Programmed Cell Death 4; *PCDH9*, Protocadherin 9; *ADAM12,* ADAM Metallopeptidase Domain 12; *CPEB1*, Cytoplasmic Polyadenylation Element-Binding Protein 1; *PARVA*, Parvin Alpha).

### 3.3. 5-Methylcytosine (m5C)

m5C is one of the most representative post-transcriptional RNA modifications [140], and it has long been known to be present in all three kingdoms of life [141,142].

m5C was originally reported in tRNAs, rRNAs [62] and coding RNAs [143]; later, it was identified in other noncoding RNAs, thanks to technologies such as bisulfite treatment and Next-Generation Sequencing (NGS) [144,145,146].

The synthesis of m5C is catalyzed by the seven members of the NOL1/NOP2/SUN domain (NSUN) family of methyltransferases [147] or by DNA methyltransferase-2 (DNMT2) [148]. These enzymes are responsibles for the methylation of rRNAs, tRNAs [149,150,151,152,153], mRNAs [154,155,156], lncRNAs [157], vault-RNAs [158], enhancer-RNAs [145], mitochondrial tRNAMet [159] and mitochondrial 12S rRNA [160].

In vitro and in vivo studies have demonstrated that aly/REF nuclear factor (ALYREF) is a putative “reader” of m5C sites on mRNAs and that, following the knockdown of NSUN2, ALYREF loses its RNA-binding ability and is retained in the nucleus, suggesting a role for m5C in mRNA exports from the nucleus [26]. A further m5C “reader” is Y-Box-Binding Protein 1 (YBX1) that recognizes and binds m5C-modified mRNAs and stabilizes their target mRNAs by recruiting ELAV-like Protein 1 (ELAVL1) [161,162].

m5C “writers” and “readers” are primarily implicated in fundamental cancer-related processes such as cell differentiation, motility [163,164], proliferation [165,166], cell cycle progression [167] and senescence [155].

In particular, NSUN2 is aberrantly expressed and plays important roles in the development and pathogenesis of different types of tumors, such as breast, colorectal, lung, skin, ovarian and bladder cancers [168].

The distribution of m5C in small RNAs is poorly understood so far; nevertheless, this modification has been recently highlighted in vault RNAs (vtRNAs) [158], piwi-associated RNAs (piRNAs) [169] and miRNAs [91,170,171]. m5C deposition regulates the processing of vault ncRNAs into small vault RNAs (svRNAs) [158,172].

m5C has been only recently characterized in miRNAs. Interestingly, methylation, but not an abundance of miR-200c-3p and miR-21-3p, was increased in pancreatic and colorectal cancer tissues, as well as in serum samples from pancreatic and colorectal cancer patients [91] (Figure 1 and Table 3).

Cheray and colleagues [170] proposed that the DNMT3A/AGO4 complex promotes the methylation of cytosine residues of miRNAs at CG dinucleotides. In glioblastoma-derived cell lines and glioblastoma tumor samples, the methylation of mature miR-181a-5p by the DNMT3A/AGO4 complex inhibits the recognition of its target mRNA BIM, a proapoptotic gene, also known as B-cell chronic lymphocytic leukemia/lymphoma (Bcl-2)-like 11 (BCL2L11) [170]. This preliminary evidence highlights that the profiling in tumor samples of m5C in miRNAs deserves further investigation.

Recently, we described that m5C is widely spread in human miRNAs in various sequence contexts by taking advantage of a novel NGS analysis of bisulfite-treated small RNAs (BS-miRNA-seq) [171].

Finally, 5mC is oxidized by the Ten-eleven translocation (TET) enzymes both in DNA and RNA. TET enzymes are Fe(II)- and 2-oxoglutarate-dependent dioxygenases that mediate the conversion of 5mC to 5hmC, then to 5-formylcytosine (5-fC) [175] and, finally, to 5-carboxylcytosine (5-caC) [18,176,177]. In DNA, these subsequent conversions have the purpose of demethylating 5mC [178], but it is still not clear if this mechanism is conserved in RNA.

hm5C has been detected in RNA isolated from different mouse and human tissues, including the brain, heart, pancreas and spleen [179]. Transcriptome-wide analyses of hm5C in mouse and in *Drosophila* RNAs have revealed the presence of 5hmC on hundreds of messenger RNAs, mainly in UC-rich motifs [180,181]. The deposition of hm5C in mRNAs has been associated with the differentiation of murine embryonic stem cells and brain development in *Drosophila* via controlling the mRNA stability or translation, respectively.

To date, decreased levels of hm5C in RNA have been shown in tumor tissues, such as CRC and hepatocellular carcinoma [176].

Interestingly, we recently unraveled not only the presence of m5C but, also, of hm5C on several miRNAs in human cancer cell lines [171]. However, no evidence of the role of hm5C modification in miRNAs in tumors has yet been reported.

### 3.4. N7-Methylguanosine (m7G)

m7G is a positively charged modification installed cotranscriptionally at the 5’ Caps of eukaryotic mRNAs [182]. This modification protects and stabilizes transcripts from exonucleolytic degradation [183] and influences all the events responsible for the processing of the mRNA molecules, from transcript elongation to translation [184,185].

Notably, the presence of internal m7G sites was found not only in tRNA and rRNA molecules [186,187,188] but also in mammalian mRNAs [188]. Internal m7G could affect mRNA translation, and this modification typically occurs near the start and stop codons in a GA-enriched motif [189].

The enzyme responsible for this internal m7G modification is METTL1, which cooperates with the cofactor WD Repeat Domain 4 (WDR4) [189,190]. Interestingly, METTL1 has been linked to tumor vascular invasion and poor prognosis in hepatocellular carcinoma [173,191].

Recently, by high-throughput screening, several miRNAs were identified as harboring internal m7G sites [192]. In particular, METTL1-dependent m7G was discovered in a subset of tumor-suppressor miRNAs involved in the inhibition of cell migration, including the let-7 family. METTL1-mediated m7G occurs on pri-miRNA within G-rich regions that display the propensity to form G-quadruplexes, i.e., structures known to be inhibitory to miRNA processing [174,193,194] (Figure 1 and Table 3).

Indeed, m7G in the let-7 family affects G-quadruplex formations, thus facilitating the formation of a canonical stem-loop structure and miRNA processing [192]. In line with this study, Liu and colleagues showed that, in colon cancer, the downregulation of METTL1 leads to a decrease in the let-7e levels. The alteration of let-7e expression affects its downstream target High Mobility Group AT-hook 2 (HMGA2), thus promoting cell proliferation, invasion and EMT [195].

## 4. Epitranscriptomic Modifications of miRNA Targets

### 4.1. m6A in miRNA Targets

The installation of m6A in miRNA targets may affect the pairing with miRNAs, thus affecting miRNA functions. Such a mechanism was first suggested in 2015, when Ke and colleagues [72] reported that the majority of m6A sites on mRNAs were in the last exon. The authors also reported a significant overlap between the m6A residues and AGO-binding sites. This finding was further corroborated by a later study [196].

Importantly, m6A modification may positively or negatively affect miRNA–mRNA pairing by several distinct mechanisms. On the one hand, m6A modifications within miRNA-binding sites may directly affect the miRNA:mRNA duplex stability. On the other hand, m6A modifications nearby AGO-binding sites may alter the mRNA secondary structure and/or recruit other RNA-binding proteins, thus modifying the mRNA accessibility to miRNA. Thus far, a few examples have been reported.

In the liver, YAP is a target of miR-582-3p, which binds at residues 313-321 of YAP 3′ UTR. Such binding is enhanced by m6A modifications of YAP 3′ UTR at residue 355. In HCC, the m6A modification at residue 355 of YAP 3′ UTR is impaired, resulting in the loss of YAP repression by miR-582-3p [197].

In cancer cell lines, m6A modification promotes the recruitment of IGF2BP1 onto Serum Response Factor (SRF) mRNA 3′ UTR. Interestingly, IGF2BP1 acts as a m6A “reader”, and its recruitment is instrumental to reduce miRNA-mediated AGO binding to SRF. Therefore, m6A deposition on the 3′ UTR of SRF mRNA relieves the negative post-transcriptional regulation by miRNAs [198]. Furthermore, a genome-wide analysis of m6A in glioma stem cells highlighted that m6A deposition on the 3′ UTR of several mRNAs may affect their targeting by different miRNAs [199]. On the other hand, Cheng and colleagues [200] showed an effect at odds with this. Indeed, in neuroblastoma, m6A modification in N-Myc 3′ UTR near a miR-98-binding site is necessary to promote the miR-98-mediated post-transcriptional repression of N-Myc. Overall, these examples highlight that the effect of m6A within mRNA 3′ UTRs on miRNA targeting is ambiguous.

m6A not only affects miRNA:mRNA interactions but, also, miRNA:ceRNA interactions. Yang and colleagues [201] showed that the m6A modification of linc-RNA-1281 is critical for its interaction with miRNAs belonging to the let-7 family. In nasopharyngeal carcinoma, lncRNA FAM225A acts as a ceRNA, sequestering miR-590-3p and miR-1275, thus activating FAK/PI3K/Akt signaling; in this scenario, m6A modification contributes to this mechanism by increasing the lncRNA FAM225A stability [202]. Finally, LINC00958, a ceRNA acting to sponge miR-3619-5p in HCC, is also stabilized through m6A modification by METTL3 [203].

### 4.2. A-to-I Editing in miRNA Targets

A-to-I editing can influence microRNA targeting. Indeed, when A-to-I editing occurs within the miRNA-binding site in the 3′ UTR of a mRNA, this can remodulate the interactions between miRNA and mRNA in different ways.

The editing of the 3′ UTR of Rho GTPase Activating Protein 26 (ARHGAP26) by ADAR1 blocks the interaction with miR-30b-3p and miR-573 to favor the expression of the protein [204]. On the contrary, in HCC cells, for instance, the RNA editing catalyzed by ADAR1 of 3′ UTR of Aryl hydrocarbon Receptor (AhR) creates a miR-378-binding site to negatively regulate the expression of this protein [205]. Through a similar mechanism, editing of the 3′ UTR of the mRNA encoding for the tumor suppressor Phosphatase and Actin Regulator 4 (PHACTR4) mediated by ADAR1 prevents the binding of miR-196a-3p. Accordingly, the decreased activity of ADAR1 in gastric cancer results in the repression of PHACTR4 by miR-196-3p [206].

Interestingly, a pan-cancer RNA editing study highlighted that the 3′ UTR of the Mouse double minute 2 homolog (MDM2) oncogene underwent A-to I editing in 11 out of the 14 cancer types investigated within a region of the 3′ UTR complementary to the miR-200 “seed” region. This editing impaired MDM2 repression by miR-200 [207].

The above-mentioned examples suggest that A-to-I editing within regions of 3′ UTR pairing with miRNA “seed” affects miRNA binding. However, a genome-wide report pinpointed that the editing of A residues lying outside of the miRNA:mRNA pairing region affects the mRNA structure, thus modulating the accessibility to AGO2-miRNA complexes [208].

### 4.3. m5C in miRNA Targets

To date, little is known about the mechanisms through which m5C can regulate the function of miRNAs, but, interestingly, a possible role for m5C in miRNA targeting was suggested by the overlap between the AGO2-binding sites and m5C positions reported in the 3′ UTR of human mRNAs [209].

## 5. Methodological Challenges and the Potential Limits of Current Knowledge

Our understanding of the mechanisms underlying the epitranscriptomic regulation of miRNAs is still potentially biased by the relatively small number of modifications which have been widely investigated in miRNAs, with most reports focused on m6A and A-to-I editing. Indeed, further mechanisms through which epitranscriptomic modifications may affect miRNA function are conceivable. For example, the recent report by Konno and colleagues suggested that m5C modification in position 9 of miR-200-3p might affect the interaction with AGO proteins [91]. However, further investigation will be required to assess whether this may represent a general paradigm. Epitranscriptomic modifications might also modulate miRNA half-life, or control miRNA subcellular localization (either in membrane enclosed organelles, or by liquid-liquid phase separation) or secretion of modified miRNAs. Secreted miRNAs have been widely reported as potential biomarkers for a variety of diseases and they also serve as signaling molecules to mediate cell-cell communications [210,211].

Our current knowledge of the modifications installed on miRNAs is likely incomplete because of technical limits which still prevent application of several high-throughput NGS methods for the quantification and characterization of epitranscriptomic modification to miRNAs. Indeed, most epitranscriptomic modifications are investigated through NGS methods which exploit the block of reverse transcription at the modified nucleobase of interest by means of different biochemical treatments (i.e., antibody cross-linking or chemical modifications). These protocols result in reads truncated at the modified positions, thus allowing the genome-wide mapping of the epitranscriptomic modification at single-nucleotide resolution [212,213]. Those methods cannot be applied to mature miRNAs, as premature reverse trascriptase termination would yield reads too short to be effectively mapped on the genome. Currently, single-nucleotide resolution analysis in miRNAs is only possible for those modifications assessed through methods based on mismatched nucleotides introduced during the reverse transcription step. Furthermore, techniques relying on unmodified nucleobases conversion (e.g., bisulfite treatment) require dedicated pipelines for data analysis as alignment of short reads is severely affected by complexity reduction associated with those techniques [171].

Finally, emerging methods to assess epitranscriptomic modifications through third generation sequencing are best-suited for long RNA molecules and cannot be easily adapted to the study of miRNAs [214].

## 6. Conclusions

In this review, we summarized the current knowledge on the epitranscriptomic modifications of miRNAs that play a role in cancer development and/or progression. In most cases, epitranscriptomic modifications exert their effect on miRNAs by affecting either their biogenesis or their binding to target mRNAs.

The current lack of mature, high-throughput technologies to profile epitranscriptomic modifications of miRNAs, with the notable exception of A-to-I editing, is likely one of the main reasons why the prognostic/diagnostic use of the “miRNA epitranscriptome” has been poorly explored thus far. Nevertheless, a plethora of studies focused on the mechanistic role of specific modifications in cancer development and/or progression point out how promising the miRNA epitranscriptome is.

Adaptation of the existing NGS methods to yield techniques allowing cheap, high-throughput and quantitative assessments of epitranscriptomic modifications in miRNAs could potentially propel research in this field by allowing mechanistic investigations of further modifications. Furthermore, such methodologies would be fundamental to investigate the potential prognostic role of miRNAs, including miRNA secreted into extracellular fluids.

## Figures and Tables

**Table 3:** Effects of m5C and m7G modifications of miRNAs in cancer.

Cancer	Modified miRNA(s)	Increase/ Decrease ^1^	Effects on miRNA Processing/Function	Effects on Tumor Progression	Reference
Glioma	miRNA-181a-5p (m5C)	↑	Binding to targets	Cytosine-methylated miRNA-181a-5p loses its ability to target the mRNA of the pro-apoptotic protein BIM	[169]
Colorectal cancer; gastric cancer; pancreatic cancer	miR-200c-3p miR-21-3p (m5C)	↑	Binding to targets	n.d.	[88]
Lung cancer	let-7 family (m7G)	n.d.	processing	m7G methylation within miRNAs regulates cell migration	[173]
Colon cancer	let-7e (m7G)	↓	processing	Down-regulation of mature let-7e results in the activation of its targets HMGA2 thus stimulating colon cancer cell viability and mobility	[174]

^1^ Increase (↑) or decrease (↓) of epitranscriptomic modifications (n.d., not detected; *HMGA2* High Mobility Group AT-hook 2).
